# Hetero-fertilization together with failed egg–sperm cell fusion supports single fertilization involved in *in vivo* haploid induction in maize

**DOI:** 10.1093/jxb/ery177

**Published:** 2018-05-11

**Authors:** Xiaolong Tian, Yuanxin Qin, Baojian Chen, Chenxu Liu, Lele Wang, Xingli Li, Xin Dong, Liwei Liu, Shaojiang Chen

**Affiliations:** 1 National Maize Improvement Center of China, College of Agronomy and Biotechnology, China Agricultural University, Yuanmingyuan West Road, Haidian District, Beijing, China; 2 Chongqing Academy of Agricultural Sciences, Jiulongpo District, Chongqing, China

**Keywords:** Cell fusion, fertilization recovery, haploid, hetero-fertilization, maize, single fertilization

## Abstract

*In vivo* doubled-haploid technology is widely applied in commercial maize breeding programs because of its time-saving and cost-reducing features. The production of maize haploids primarily depends on the use of Stock6-derived haploid inducer lines. Although the gene underlying haploid induction, *MTL/ZmPLA1/NLD*, was cloned recently, the mechanism of haploid induction is still unknown. Hetero-fertilization can occur via a single fertilization, which provides a means to investigate single-fertilization events by studying the hetero-fertilization phenomenon. In this study, we found that the hetero-fertilization rate increased significantly when female maize lines were first individually crossed with pollen from the inducer CAU5 in dual-pollination experiments 4 h before a second pollination with common lines. We also examined embryogenesis during haploid induction by confocal laser-scanning microscopy and observed single-fertilized ovules, indicating that single fertilization occurred during haploid induction. We therefore postulate that both single fertilization and chromosome elimination contribute to haploid induction in maize. We also propose a scheme for the formation of hetero-fertilized and haploid kernels. Our results provide an efficient approach to identify hetero-fertilized kernels for research on interactions between embryo and endosperm.

## Teaser

Hetero-fertilization and failed egg–sperm cell fusion indicate that single fertilization plays a key role in *in vivo* haploid induction in maize.

## Introduction

Enhancement of hybrid crop vigor by outbreeding of parent strains, i.e. heterosis, contributes greatly to the security and abundance of the worldwide food supply. Maize is a crop for which hybrid vigor has been much improved over the years, in part by the production of homozygous lines. Given the rapidly increasing human population, limited land resources, and the changing climate throughout the world, rapid and efficient breeding strategies that can maximize the rate of gain from genetic selection are needed. In recent decades, commercial maize breeding strategies have often used doubled-haploid (DH) technology to develop homogeneous lines. Compared with conventional recurrent-selfing methods, which usually require 6 to 10 generations to produce a homozygous line, DH technology needs only two generations, thereby saving time and reducing costs. By pollinating the maternal source germplasm with pollen from a paternal inducer, the ears obtained carry a proportion of kernels that contain haploid embryos. Currently, to discriminate haploid kernels from crossed-diploid kernels, pigmentation induced by the encoded protein of the dominant pigmentation maker gene *R1-nj* carried by the inducer is normally used. Purple anthocyanin pigmentation on the scutella and aleurones of the crossed-diploid kernels is usually found as a consequence of *R1-nj* expression, whereas haploid kernels have a colorless scutellum and pigmented aleurone. The color difference allows for easy visual discrimination and separation of the haploid and diploid kernels. However, classification of seeds by the color of their scutella and aleurones can be undermined by expression of pigmentation inhibitor alleles, e.g. *C1-I* (Paz-Ares *et al.*, 1990), in the genomes of some targeted maternal germplasms—especially those of flint and tropical germplasms—so that widespread application of DH technology based on expression of *R1-nj* has been limited. Instead, the oil-content (OC) classification system has been proposed as an alternative way to discriminate between haploid and diploid kernels without risk of interference from the genetic backgrounds of the targeted germplasms. Chen and Song (2003) showed that crossed-diploid maize kernels had an OC that was 30% greater than that of haploid kernels from ears pollinated by the high oil-inducer CAUHOI, and Rotarenco *et al.* (2007) showed that diploid maize kernels produced an average of 19.4% more oil than haploid seeds. Based on experimental results, Melchinger *et al.* (2013, 2014) reconfirmed that the use of OC to identify diploid and haploid kernels is efficient, generally applicable, and can be used for high-throughput identification of diploid versus haploid kernels.

To date, almost all *in vivo* maize haploid inducers are derived from the same ancestor line, Stock6 (Hu *et al.*, 2016). Recently, the Stock6-derived gene conferring haploid induction was cloned by three independent groups in the USA, China, and France, and named *MATRILINEAL* (*MTL*), *ZmPHOSPHOLIPASE A1* (*ZmPLA1*), and *NOT LIKE DAD* (*NLD*), respectively (Gilles *et al.*, 2017; Kelliher *et al.*, 2017; Liu *et al.*, 2017*b*). This gene encodes a patatin-like phospholipase A, which is expressed in mature pollen. A 4-bp insertion in the last exon causes a frameshift mutation and the early truncation of the encoded protein. Gilles *et al.* (2017) predicted that loss of the C-terminal lipid-anchor domain in the truncated inducer protein would cause its mislocalization in the pollen, leading to a defective fusion of the sperm cell and female gamete, and resulting in haploid formation. Although the causative gene has been found, the mechanism underlying the haploid formation is still unclear.

Single fertilization and chromosome elimination are two models that have been hypothesized to explain *in vivo* haploid formation in maize. In the first model, one sperm cell from the inducer gamete fails to fuse with the egg cell but triggers haploid embryogenesis. In the second model, two sperm cells from a single inducer gamete fuse with the female gamete, but the chromosomes from the inducer are eliminated during subsequent cell divisions. The second model has more evidence to support it, including observations of micronuclei in part of the ovules fertilized by inducer pollen and the presence of a rare inducer DNA fragment detected in certain haploid seeds (Li *et al.*, 2009; Zhao *et al.*, 2013; Qiu *et al.*, 2014). Zhao *et al.* (2013) generated the inducer line CAU^B^, which contains B chromosomes, to pollinate the hybrid ZD958 maize line. A few progeny haploid embryos containing B chromosomes were detected, providing evidence for a selective chromosome elimination mechanism. Nevertheless, considering that most of the generated haploids did not contain a B chromosome, the possibility that other mechanisms contributed to *in vivo* haploid induction could not be excluded. Conversely, robust evidence that supports the single-fertilization model is still absent.

In maize, two sperm cells from a single pollen grain usually fuse with the two female gametes, the egg cell and the central cell, to form the embryo and endosperm, respectively. Any defect in double fertilization will result in lethal or abnormal kernels. Hetero-fertilization (HF) occurs when the egg cell and central cell are fertilized by sperm cells from different pollen grains. Haploid induction represents a process during which pleiotropic phenotypes, e.g. embryo-aborted kernels and endosperm-aborted kernels, are closely associated with haploid formation (Dong *et al.*, 2013; Xu *et al.*, 2013). These abnormal kernels indicate aberrant double fertilization, and embryogenesis may occasionally occur during haploid induction. A fertilization-recovery mechanism has been discovered that allows the restoration of ovule fertility in Arabidopsis after the initial failure of the double fertilization (Beale *et al.*, 2012; Kasahara *et al.*, 2012; Sprunck, *et al.*, 2012; Maruyama *et al.*, 2013). By attracting and accepting the second pollen tube with the second/persistent synergid cell, Arabidopsis ovules compensate for the failure of the first fertilization, thus guaranteeing the success of reproduction.

The ability to link the HF phenomenon with a fertilization-recovery mechanism allows us to consider that when a single fertilization occurs, HF may also occur owing to its linkage to the fertilization-recovery mechanism (Maruyama *et al.*, 2013), which would allow for the indirect investigation of single-fertilization mechanisms by studying HF events in maize. HF in maize was first observed by Sprague (1929) and was shown a few years later to originate in ovules fertilized by sperm from different pollen grains (Sprague, 1932). The spontaneous HF rate in maize has been reported to be, on average, <2% (Sprague, 1932; Gao *et al.*, 2011), but the rate varies according to the genetic background of the maternal lines. Kato (1997) applied a dual-pollination procedure by first pollinating maize ears with a bicellular pollen, which contained only one sperm cell and one vegetative nucleus, then pollinating with pollen from the *R1-scm2* line, and this generated haploid and HF kernels. The results of this experiment suggested that a single fertilization could result in haploid and HF seeds.

In this study, we applied dual pollinations using the haploid inducer line CAU5 or the non-inducer line B73*R1-nj* (BR) and the high-OC line GY923 to generate HF kernels and to determine their HF rates, which would reflect their single-fertilization rates. We also verified the usefulness of the method that has been developed to identify a HF or haploid kernel based on its OC. In addition, factors potentially affecting double fertilization and the embryogenesis process during haploid induction were investigated. Finally, we propose a scheme for haploid and HF formation. Collectively, this study increases our knowledge of the mechanisms underlying *in vivo* haploid induction and double fertilization in maize.

## Materials and methods

### Plant material and pollination

**Fig. 1.**
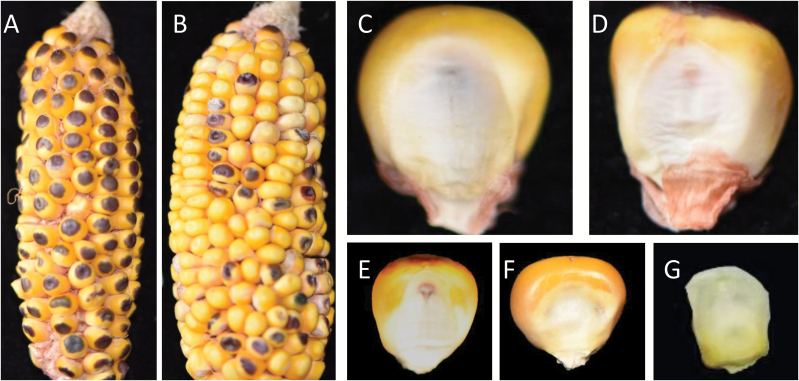
Diversity of kernel types formed during haploid induction. (A) Kernels on an ear singly pollinated with the inducer CAU5, showing substantial *R1-nj* pigmentation. (B) Kernels on an ear pollinated by the high-oil content line GY923 4 h after initial pollination with the inducer CAU5. (C) A kernel from a type-1 hetero-fertilization (HF) showing a colored scutellum and a colorless aleurone. (D) A putative haploid kernel, showing a colored aleurone and a colorless scutellum, which can be associated with haploid or type-2 HF kernels. (E) A diploid kernel from a Bo × CAU5 cross showing a colored scutellum and a colored aleurone. (F) An embryo-aborted kernel. (G) An endosperm-aborted kernel. The B73*os1* line was used as the female parent throughout.

Maize (*Zea mays*) ears were pollinated twice, once at the beginning of an experiment and then 4 h later. The pollinators were GY923, an inbred line with a mean OC of ~12%, B73*R1-nj*, a B73 near-isogenic line with an *R1-nj* marker but without haploid-induction ability (abbreviated as BR), and CAU5, an *in vivo* haploid inducer with a haploid induction rate of ~10%. The female lines were the inbred line B73*os1*, a B73 near-isogenic line (abbreviated as Bo), and five other elite inbred lines in China (8701, C7-2, Z58, J24, and Q319), which were used because the *R1-nj* pigmentation marker was well expressed in their scutella and aleurones when pollinated with the inducer CAU5 (Fig. 1A, C–E).

Initially, the crosses were performed manually at the Shangzhuang Experimental Station, Beijing, China (BJ) in the summer of 2015. We performed two different single-pollination and three different dual-pollination crosses, with the first pollination at 10.00 h and the second pollination at 14.00 h (Table 1); for example, CAU5+GY923 means we performed pollination with CAU5 pollen first and with GY923 pollen 4 h later. In general, the number of kernels in ears pollinated by an inducer is much smaller than the number pollinated by a non-inducer (Dong *et al.*, 2013; Xu *et al.*, 2013). Therefore, to guarantee that a sufficient number of ears and kernels were available for statistical analysis after pollination, we performed 20 or more crosses for each pollination experiment. The harvested ears were carefully examined kernel by kernel, and those ears with >50 induced kernels were selected for a further analysis.

**Table 1.** Single and dual pollinations used in this study

Pollinators*	First pollinator	Second pollinator
CAU5	CAU5	none
CAU5+CAU5	CAU5	CAU5
CAU5+GY923	CAU5	GY923
GY923	GY923	none
GY923+CAU5	GY923	CAU5

*The inbred line B73*os1* was used as the female in all pollination experiments.

To verify the universality of the results from the initial set of experiments, the other five lines mentioned above (8701, C7-2, Z58, J24, and Q319) were used as the female parents under the same pollination conditions. Crosses were also conducted at BJ in the summer of 2016 and at the Sanya Experimental Station at Hainan Island (HN), China, in the winter of 2016. To avoid contamination by alien pollen in the single- and dual-pollination experiments, the female parents were emasculated and their ears were covered with paper bags before silking; in addition, their silks were cut off the day before pollination.

### Kernel classification

The harvested ears were threshed, and their kernels were grouped into one of six classes preliminarily based on the pigmentation of their scutella and aleurones, as follows: (i) non-induced kernels (Bo–GY923 cross, non-pigmented scutella and aleurones); (ii) type-1 HF kernels (pigmented scutellum and non-pigmented aleurone, Fig. 1C); (iii) putative haploids (including haploids and type-2 HF kernels; non-pigmented scutella and pigmented aleurones; Fig. 1D); (iv) induced crossed-diploids (pigmented scutella and aleurones; Fig. 1E); (v) embryo-aborted kernels (Fig. 1F); and (vi) endosperm-aborted kernels (Fig. 1G). Induced kernels, as defined for this research, included the kernels in classes (ii) to (vi). We calculated the rates of kernel induction for each class as:

$$R_{\text{i}}=n_{\text{i}}/n_{\text{total}}$$

where *R*_i_ is the rate of induced kernels of a given class, *n*_i_ is the number of induced kernels of a given class, and *n*_total_ is the total number of induced kernels. Calculation of *R*_i_ for each class allowed us to assign a putative haploid-production rate (PHPR), type-1 and type-2 HF rates, and embryo- and endosperm-abortion rates (EmAR and EnAR, respectively) for further analysis.

### Classification and verification of haploid and type-2 HF kernels

After preliminary classification, the amount of OC in the putative haploid kernels (including haploid and type-2 HF kernels) and in at least 30 kernels from each of the other classes was measured using a nuclear magnetic resonance mq20 instrument (Bruker, Germany). The OC threshold used to sort the type-2 HF from the putative haploid kernels was determined with reference to the corresponding OC classification for haploids (Dong *et al.*, 2014; Melchinger *et al.*, 2014). Next, all putative haploid kernels were planted in separate plots based on their OC to verify the accuracy of their OC classifications by assessing their field performance.

### Triphenyl tetrazolium chloride staining and pollen-tube growth assays

Samples of fresh pollen were individually placed into 2-ml centrifuge tubes at 10.00 h, then 0.5 ml of a 0.5% (w/v) triphenyl tetrazolium chloride solution was added into each tube. After placing the tubes for 15 min in an incubator at 35 °C, the pollen samples were evaluated for the degree to which they were stained by examining bright-field microscopic images. The microscopy was conducted under a Leica S6D StereoZoom and pictures were taken using a Canon camera. Pollen germination assays were conducted on solid pollen-germination medium made of 1.2% (w/v) low-melting agarose and an equal volume of 100 μl 1% boric acid, 10 μl 1 M KH_2_PO_4_, 800 μl 2.5 M Cacl_2_, 25% (w/v) sucrose, and 12% (w/v) PEG 4000 in 3-cm Petri dishes. The pollen grains were cultured for 30 min at room temperature (~28 °C), then immediately photographed under a microscope, and then again 5 min later. The lengths of the pollen tubes were measured using Image-Pro Plus 6.0, and the rate of pollen tube growth during the 5-min period between photographs was determined.

### Confocal laser-scanning microscopy

Confocal laser-scanning microscopy was conducted according to Zeng *et al.* (2009) with minor modifications. Ovules at 1–6 d after pollination (DAP) were collected and fixed in 50% (v/v) ethanol/acetic acid/methanol, 90:5:5 v/v/v, for at least 24 h. The samples were then stored in 70% (v/v) ethanol at 4 °C after washing with 50% (v/v) ethanol. The ovule samples were rehydrated by serial emersion in 50%, 30%, and 10% (v/v) ethanol and then immersed in a bath of distilled water for 20 min. Next, the ovules were treated in 2% (w/v) aluminum potassium sulfate for 20 min and then washed twice with distilled water. The ovules were then stained with Eosin B [10 mg l^–1^ in 4% (w/v) sucrose solution] for 24–48 h at room temperature. The samples were then treated again with 2% (w/v) aluminum potassium sulfate for 20 min and washed twice with distilled water. The samples were serially dehydrated in 10%, 30%, 50%, 70%, 90% (v/v), and 100% ethanol for 20 min each. Finally, the dehydrated samples were transferred into a mixture of ethanol and methyl salicylate (1:1, v/v) for 1 h and then stored in pure methyl salicylate prior to observation under a Nikon laser-scanning confocal microscope. The excitation wavelength was 543 nm, and the emitted light was detected between 550 and 630 nm.

### Statistical analysis

The rates of induced kernels of a given class (*R*_i_) from single- and double-crosses, and the rates of pollen viability (High, Medium, Non-viable) were analysed by ANOVA. Student’s *t*-tests were used to determine the significance of differences in *R*_i_ between CAU5+GY923 and other single- or double-crosses.

## Results

### The Type-1 HF rate and PHPR were greater in the CAU5+GY923-pollinations than in other dual-cross pollinations

**Fig. 2.**
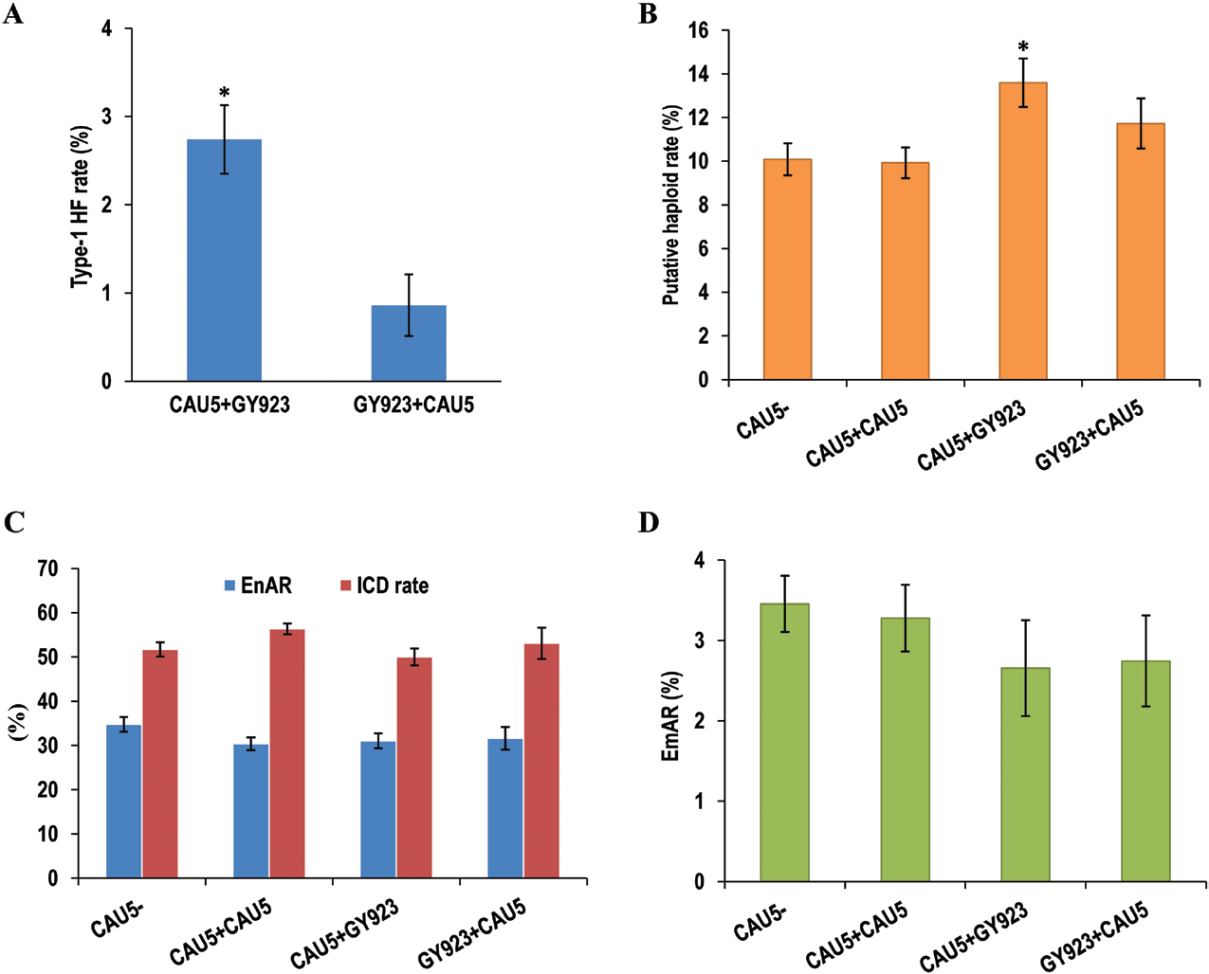
Comparison of the type-1 hetero-fertilization (HF) rate and putative haploid-production rate (PHPR) for kernels from each dual-pollination experiment. The type-1 HF rate (A) and PHPR (B) were greater when the inducer CAU5 was the first to pollinate the line B73*os1* in the dual-pollination. By undergoing type-1 HF and type-2 HF, some endosperm-aborted kernels and embryo-aborted kernels were rescued at a very early stage, which led to a decrease in the endosperm-abortion rate (EnAR) and induced crossed-diploid (ICD) rate (C), and in the embryo-abortion rate (EmAR) (D) in the dual pollinations in comparison with the single pollinations. Data are means (±SD). Significant differences from GY923+CAU5 were determined using Student’s *t*-test; *P<0.05.

Because the tassels of all maternal plants had been removed and the ears had been completely covered with paper bags prior to the emergence of their silks, almost no non-induced kernels (<1%) were present after the ears had been singly pollinated with CAU5 or doubly pollinated with the CAU5+CAU5 regime (Supplementary Table S1 at *JXB* online). The small number of non-induced kernels present could have been the result of pollen contamination in the air. Considering the very low rate of contamination (6 out of 7064, Supplementary Table S1) found for type-1 HF, we considered that the data generated in the experiment was reliable. Induced phenotypes (which includes haploid kernels, embryo-aborted kernels, and endosperm-aborted kernels etc.) were detected for all pollination regimes except for the single GY923 pollination (Supplementary Table S1), which reflected the effects of the inducer pollen. The *R1-nj* marker was well expressed in both the single- and dual-crosses (Fig. 1A, B), making it suitable for visually classifying the different kernels (see Methods section). We grouped the HF kernels into two classes according to their different scutellum and aleurone *R1-nj* pigmentations. The type-1 HF kernels had pigmented scutella and colorless aleurones (Fig. 1C), whereas for the type-2 HF kernels the scutella were colorless and the aleurones were colored. The haploid (Fig. 1D) and the type-2 HF kernels had the same color profile, which made it impossible to tell them apart visually. The results showed that the rates of type-1 HF and PHPR induced by the CAU5+GY923 dual pollination were significantly greater than those of other dual crosses (Fig. 2A, B). The mean value for the type-1 HF induction rate for the 16 ears used in the CAU5+GY923 pollination was 2.74%, which was three times greater than that found for the GY923+CAU5 pollination (0.86%, Fig. 2A). Similarly, the PHPR for the CAU5+GY923 pollination was 13.59%, which was at least 2% greater than those found for other pollinations (Fig. 2B). We attributed the greater PHPR for the CAU5+GY923 pollination to the number of type-2 HF events. Two persistent co-products of haploid induction, namely aborted endosperm and embryos (Fig. 1G, F, respectively), were also found. The EnAR seemed to be slightly less in dual-pollination than in single-pollination (35% in the single CAU5-pollination and 31% in each dual-pollination; Fig. 2C), although the differences were not significant. This tendency was also observed for the EmAR (Fig. 2D) in dual-pollination, although the rate values were much smaller than those observed for EnAR. Collectively, these data indicated that the total number of HF kernels found in the CAU5+GY923 pollination may have compensated for the number of aborted kernels, leading to increases in the induced crossed-diploid rates (Fig. 2C) and decreases in the endosperm- and embryo-abortion rates.

### Detection and verification of type-2 HF kernels

**Fig. 3.**
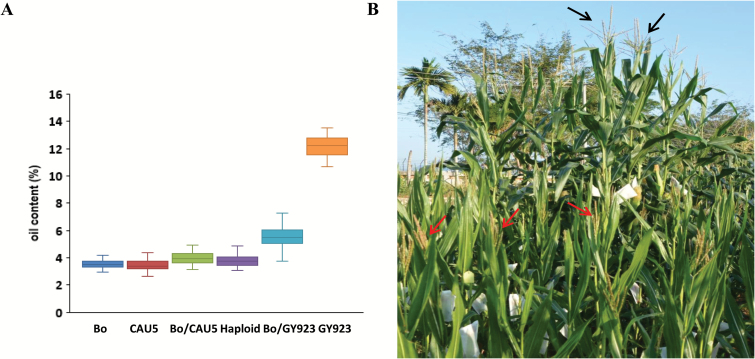
Identification and verification of type-2 HF kernels. (A) Variation in the oil content (OC) in kernels formed in the single- and dual-pollination experiments. (B) Field performance of haploid plants (red arrows) and type-2 HF plants (black arrows).

We assumed that the increase in the PHPR found in the CAU5+GY923 pollinations was a consequence of an increase in the type-2 HF rate; however, it was impossible to distinguish type-2 HF and haploid kernels according to the anthocyanin pigmentation in their scutella and aleurones. To discriminate haploid from crossed-diploid seeds, previous studies have proposed using xenia effects related to the OC in the kernels (Chen and Song, 2003; Rotarenco *et al.*, 2007; Melchinger *et al.*, 2013), which is similar to the way we sorted type-2 HF kernels from putative haploids in this research. In our experiments, haploid and type-2 HF kernels had the same endosperm genotype, whereas the embryo genotypes were substantially different. Usually, the haploid embryos only retained the maternal genotype (Bo), whereas the type-2 HF embryos had a heterozygous genotype (Bo/GY923). Expression of GY923-type genes caused the type-2 HF kernels to have a large OC, which enabled us to easily separate type-2 HF and putative haploid kernels. GY923 is a high-oil inbred line, with an OC between 10 and 14%, whereas common lines have an OC between 2% and 5% (Fig. 3A). No significant differences were detected between the OC of Bo, CAU5, the diploid Bo/CAU5, and haploid kernels that were produced by crosses. For ovules fertilized by GY923 pollen (Bo/GY923), the progeny had a significantly increased OC (Fig. 3A). Considering that type-2 HF and diploid Bo/GY923 embryos shared the same genotype, the type-2 HF embryos might have been expected to have a similar variation in OC. The maximum OC of the haploids generated in the CAU5 single pollination was <5%, meaning that in the C5+GY923 and GY923+C5 experiments putative haploids with an OC>5% would probably be putative type-2 HF kernels. The OC of putative haploid seeds was quantified by NMR spectroscopy, and then classified as either OC>5% or OC≤5%. After separating the seeds into these two groups, we planted 100 of the putative type-2 HF kernels with an OC>5% and 200 of the putative haploid kernels with an OC≤5% in two separate field plots. The ploidy status of the mature putative haploid plants was confirmed by field performance. The haploid plants were short, with small tassels, and erect and narrow leaves, whereas the type-2 HF plants displayed hybrid traits, i.e. they were tall and strong (Fig. 3B). At the end of the experiment, 268 of the plants were finally verified for ploidy status, with 90 being classified as type-2 HF plants and 178 as haploid plants (Table 2). Of the putative type-2 HF kernels, 94.44% were verified to be HF kernels, whereas of the putative haploid kernels 15.73% were proved to be type-2 HF kernels, which suggested that the type-2 HF rate in the C5–GY923 experiment was >2.96% (75 out of 2538; Supplementary Table S2). In addition, the results indicated that the type-2 HF and haploid kernels could be effectively separated based on their OC.

**Table 2.** Characterization of putative type-2 HF kernels according to their oil content

Kernel classification before OC analysis	OC*	*n*	True haploid kernels	True type-2 HF kernels (%)
Putative type-2 HF	>5%	90	5	85 (94.44)
Putative haploid	≤5%	178	150	28 (15.73)

*OC, oil content.

### HF and haploid formation in maize of different genetic backgrounds

In the summer of 2015, we harvested more than 5.71% type-1 and type-2 HF kernels from the CAU5+GY923 pollination experiment but only 2.41% HF kernels of both types from the GY923+CAU5 pollination experiment. To verify the universality of the substantial increase in HF kernels associated with the CAU5+GY923 pollination, different types of maternal germplasms were subjected to CAU5+GY923 pollination. Five maternal inbred lines with different genetic backgrounds that expressed high levels of *R1-nj* were individually pollinated by CAU5+GY923 and BR+GY923. The putative haploid and putative type-2 HF kernels were sorted according to their pigmentation first and then according to their OC, and were subsequently verified by their plant performance. Given that OC measurements and germination rates in the field are very accurate methods of classification (Supplementary Table S3), we used these criteria to determine the putative HF rate (PHFR) for further analysis. The results of the experiments performed in 2016 in BJ and HN showed that the PHFR for the CAU5+GY923 pollination was much greater than for the BR+GY923 experiment (Table 3). For the CAU5+GY923 experiments in BJ, the PHFR ranged from 1.50% to 5.68%, whereas the PHFRs for the BR+GY923 experiments were all <1% (between 0.20% and 0.88%; Table 3). Similarly, in 2016, the PHFR for the CAU5+GY923 experiments in HN ranged from 1.44% to 6.14%, whereas the rates for the BR+GY923 experiments were only between 0.84% and 1.38% (Table 3). Our results demonstrated that the PHFR could be increased by dual pollination with an inducer as the first pollinator regardless of its genetic background, and that the PHFR was affected by the maternal genotype as well as by environmental factors, which was also true for the haploid rate (HR). In addition, it seemed that maternal germplasms with large HR have a greater potential to produce HF kernels (Table 3), suggesting that additional maternal material should be tested for their PHFR and HR to determine a correlation between the two rates. Collectively, our data implied that single fertilization was involved in haploid induction, as the HF rate was greater in dual crosses when the female parent was first pollinated by inducer pollen.

**Table 3.** Putative hetero-fertilization and haploid rates after pollination for a diverse group of male and female germplasms

Female	Pollination	2016 BJ			2016 HN		
		PHFR (%)	HR (%)	N	PHFR (%)	HR (%)	N
8701	BR+GY923	0.20	0	1050	1.38	0	434
	CAU5+GY923	5.68	8.42	582	6.14	10.68	440
C7-2	BR+GY923	0.58	0	2060	0.84	0	1424
	CAU5+GY923	1.50	4.32	2266	1.44	6.62	2159
Z58	BR+GY923	0.36	0	2468	1.05	0	859
	CAU5+GY923	3.84	7.47	1900	1.69	4.83	829
J24	BR+GY923	0.86	0	1168	1.06	0	661
	CAU5+GY923	3.06	6.80	588	2.38	3.36	421
Q319	BR+GY923	0.88	0	113	1.26	0	715
	CAU5+GY923	2.54	8.24	490	2.60	8.95	962

BJ, Shangzhuang Experimental Station, Beijing; HN, Sanya Experimental Station, Hainan Island; PHFR, putative hetero-fertilization rate; HR, haploid rate; N, the number of total induced kernels.

### Pollen viability and germination

**Fig. 4.**
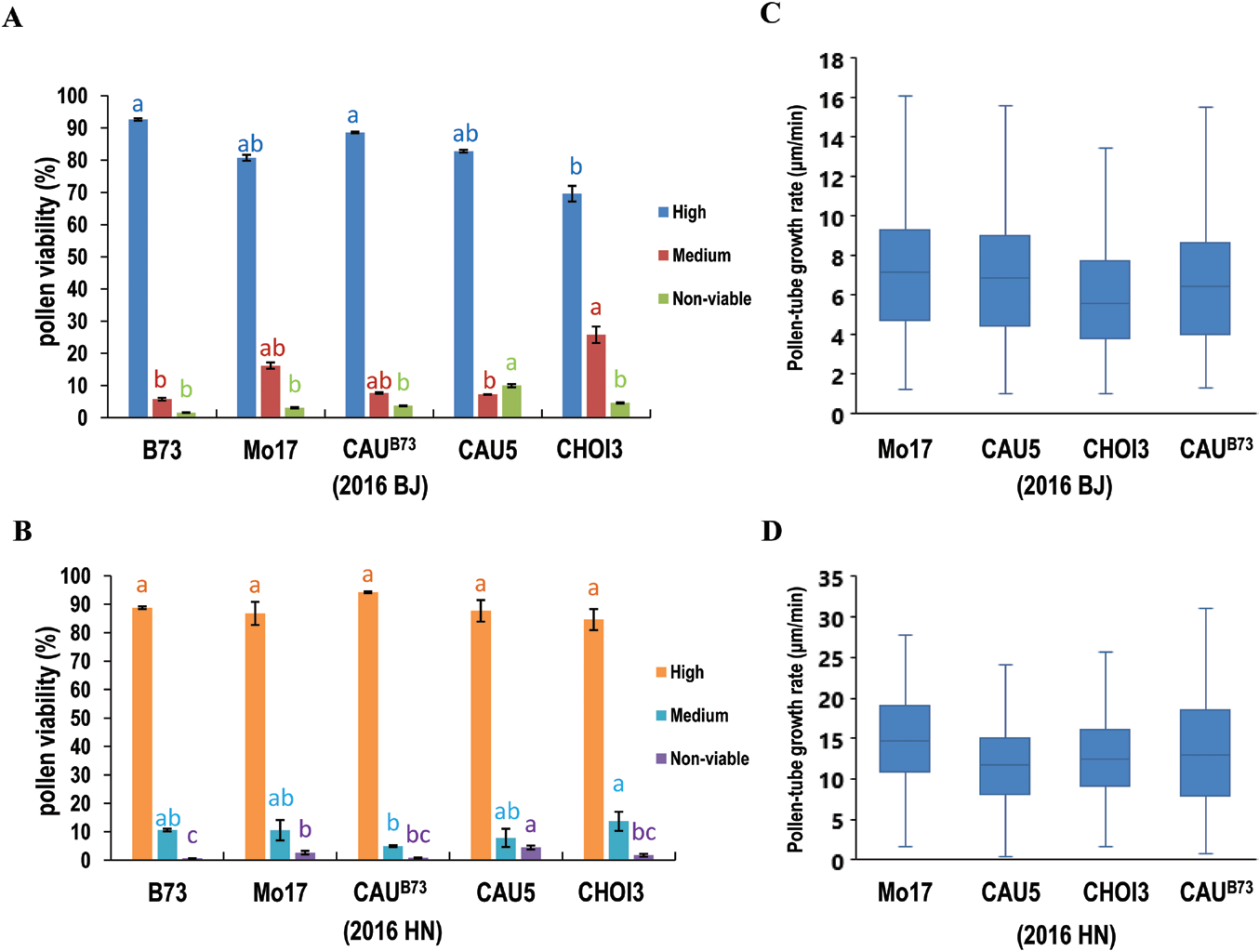
Comparison of pollen viability and pollen-tube growth rates associated with inducer and non-inducer pollen grains. No significant differences were found for pollen viability (A, B) and the growth rates (C, D) associated with inducer and non-inducer pollen grains used for cultivation in the 2016 (A, C) and HN (B, D) field tests conducted at Shangzhuang Experimental Station, Beijing, and Sanya Experimental Station, Hainan Island, respectively. Different letters in (A) and (B) indicate significant differences according to the results of multiple comparisons (*P*<0.05). Data are means (±SD) in (A) and (B). The box-plots in (C) and (D) show the mean, the first and third quartiles, and the maximum and minimum values.

The types of pleiotropic phenotypes most associated with haploid formation indicate that aberrant double fertilization may occur during *in vivo* haploid induction (Gilles *et al.*, 2017; Kelliher *et al.*, 2017; Liu *et al.*, 2017*a*). A defect in germline development, pollen-tube growth, or gamete fusion will result in an aberrant double fertilization (Dresselhaus *et al.*, 2016; Zhou *et al.*, 2017). To investigate the factors affecting double fertilization during *in vivo* haploid induction, we first looked for aberrant development of the CAU5 germline; however, none of the examined pollen grains showed a single sperm cell (data not shown). We then examined the pollen viability of three inducer lines, CAU5, CHOI3, and CAU^B73^, as well as two inbred non-inducer lines, B73 and Mo17, which served as controls. The pollen grains were classified according to the intensity of their triphenyl tetrazolium chloride staining, i.e. as high viability, medium viability, and non-viable. The CAU5 pollen had the greatest percentage of non-viable pollen grains, whereas the other two inducer lines and the two inbred lines possessed similar percentages of non-viable pollen grains (Fig. 4A, B). It appeared that the inducer and inbred lines had a similar percentage of pollen grains with medium viability, implying that the extent of haploid induction during double fertilization was not affected by the viability of the inducer pollen. The phospholipase encoded by the haploid-induction gene has been reported to be critically responsible for pollen-tube growth in Arabidopsis (Kim *et al.*, 2011), which lead us to examine the *in vitro* pollen-tube growth rates of the three inducers. The pollen-tube growth rate of Mo17 was also measured as a control. The CHOI3 pollen (from the 2016 BJ and HN field experiments) had a slower growth rate than that of Mo17, CAU5 pollen (from the 2016 HN field experiment) had a slower tube growth than that of Mo17, whilst no significant difference was detected between the tube-growth rates of CAU^B73^ and Mo17 (Fig. 4C, D). Collectively, these data demonstrated that not all inducers had pollen with an abnormal viability or tube-growth rate, suggesting that the reproductive defect that leads to haploid induction may occur during later the fusion processes of the gamete cells or during subsequent cell divisions.

### Embryogenesis involved in haploid induction

**Fig. 5.**
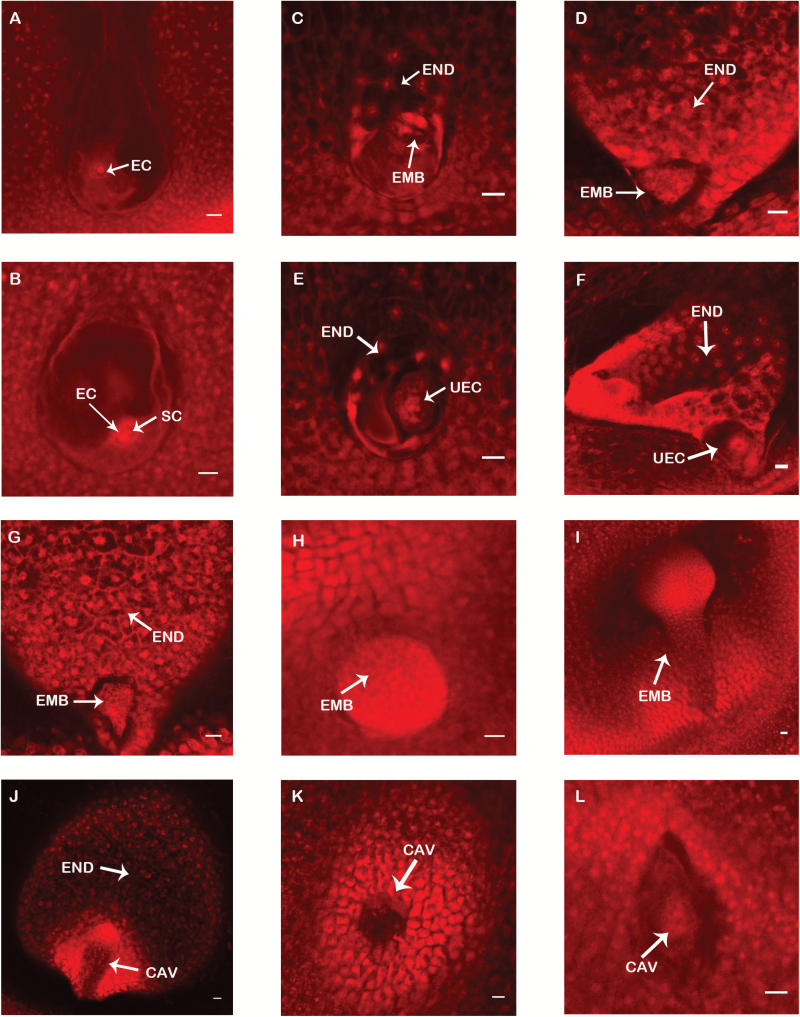
Single fertilizations occur during haploid induction. (A–L) Embryogenesis during haploid induction from 1 to 6 d after pollination (DAP). (A) Unfertilized ovule. (B) At 1 DAP, cell fusion has begun. (C) At 2 DAP, cell divisions occur in doubly fertilized ovules. (D) At 3 DAP, the young embryo has formed in doubly fertilized ovules. (E) At 2 DAP, some ovules contained an unfertilized egg cell and a fertilized central cell. (F) At 3 DAP, the egg cell has not fused with sperm cells in single fertilized ovules. (G–I) Development of doubly fertilized ovules between 4 and 6 DAP. (J–L) Aberrant development of singly fertilized ovules between 4 and 6 DAP. EC, egg cell; SC, sperm cell; EMB, embryo; END, endosperm; UEC, unfertilized egg cell; CAV, cavity. A cavity forms in the embryo region when the embryo has not developed properly. Scale bars are 20 μm.

To explore the double fertilization and embryogenesis processes involved in haploid induction, we examined the development of ovules from B73 ears pollinated by the inducer CAU5 between 1 and 6 DAP using confocal laser-scanning microscopy. Some unfertilized ovules were always observed on each day (Fig. 5A). At 1 DAP, we observed that cell fusion had begun in most ovules (Fig. 5B). The fertilized central cells (endosperm) and zygotes (embryos) in most ovules had undergone a few divisions by 2 DAP (Fig. 5C); however, in some ovules, although the fertilized central cells had divided a few times, it appeared that the associated egg cells had not been fertilized (Fig. 5E, Supplementary Fig. S1). Doubly fertilized ovules developed normally thereafter (Fig. 4D, G–I), whereas fertilized ovules with egg cells that were still not fertilized by 3 DAP (Fig. 5F) developed abnormally (Fig. 5J–L), and might have eventually aborted to form various types of defective kernels (Xu *et al.*, 2013). Taken together, our results showed that single fertilizations, i.e. a fertilized central cell and unfertilized egg cell, occurred during *in vivo* haploid induction and could explain the origin of increased HF in our dual-pollination experiments. However, ovules with developed embryos alone were not observed, and hence direct evidence for the contribution of single fertilization to the formation of haploid and HF kernels is still required. Future experiments involving special maternal lines with high inducibility and inducers with fluorescent germ cells may help to further characterize double fertilization and later embryogenesis events during haploid induction.

## Discussion

### A scheme for formation of haploid and hetero-fertilized kernels in maize

In recent decades, a major development in commercial maize breeding has been the widespread application of doubled haploid (DH) technology based on *in vivo* haploid induction. Haploid-inducer lines share the same ancestor. The gene conferring haploid formation was cloned recently but the mechanism of haploid induction is still unknown. Observations of haploids with a B chromosome or inducer DNA fragment have been explained by the incomplete paternal inducer-chromosome-elimination model (Li *et al.*, 2009; Zhao *et al.*, 2013; Qiu *et al.*, 2014), although other mechanisms, such as single fertilization, may also be involved.

Sperm cells of an angiosperm are delivered to the egg apparatus (egg cell and accessory synergid cells) via the pollen tube. Upon arrival at the ovule, the pollen tube communicates specifically and precisely with the receptive synergid cell, so that the cargo can be released towards the cleft between the egg and central cells. After successful positioning, adhesion, and activation of the sperm cells, fusion of female gametes and two sperm cells occurs, forming a diploid embryo and triploid endosperm, which completes the double fertilization (Dresselhaus *et al.*, 2016). However, single fertilization may occur if one of the two sperm cells is defective (Iwakawa *et al.*, 2006; Kim *et al.*, 2008). Flowering plants have developed certain mechanisms, such as polyspermy block and fertilization recovery, to ensure successful double fertilization. Polyspermy block prevents multiple pollen tubes from being attracted to the embryo sac; conversely, the second synergid cell functions to attract another pollen tube, allowing double fertilization to be completed if the first fertilization has been defective (Beale *et al.*, 2012; Kasahara *et al.*, 2012; Sprunck, *et al.*, 2012; Maruyama *et al.*, 2013). Qiu *et al.* (2014) observed fertilized ovules in a HZ514 (non-inducer) × HZI1 (haploid inducer) cross using the whole stain-clearing technique. They found that sometimes a sperm cell (or cells) could still be observed remaining in the synergid cells while the young embryo and endosperm had already divided, indicating that fertilization recovery had occurred and that both the synergid cells may play a critical role during haploid induction.

The single fertilization model has long been proposed to explain haploid formation; however, robust evidence has always been lacking due to the difficulties involved in observing the maize double fertilization process. Fortunately, the fertilization recovery mechanism and hetero-fertilization provide a means to investigate single fertilization. In this study, we determined the presence of single fertilization during haploid induction, and we propose a scheme for haploid and HF formation based on our results and those of previous research.

**Fig. 6.**
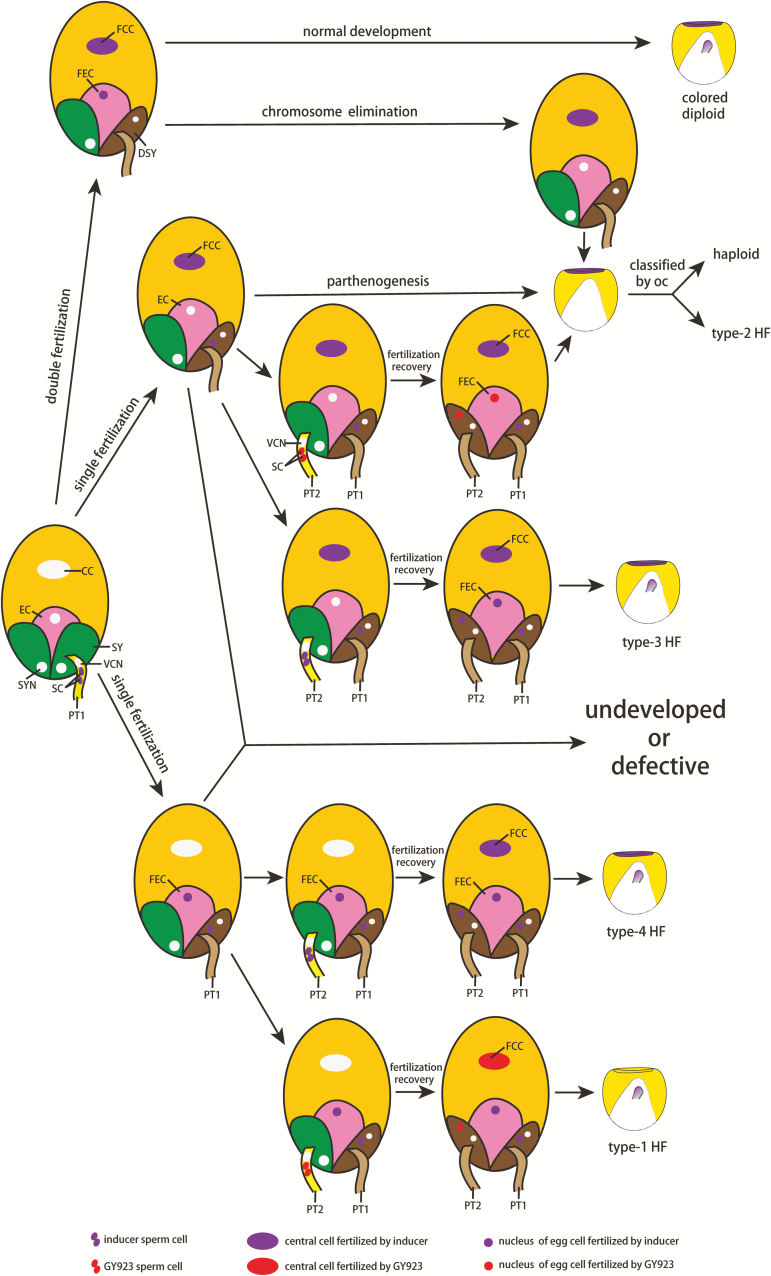
Proposed scheme for haploid and hetero-fertilization (HF) formation during dual pollination. Most ovules doubly fertilized by inducer sperm cells will develop into colored diploid kernels, although chromosome elimination may occur in the zygotes of other ovules leading to formation of haploid kernels. Singly fertilized ovules often may not fully develop, or produce defective kernels. In some cases, the central cells of ovules will only be fertilized by an inducer sperm cell, and the ovules will develop into haploid kernels by parthenogenesis. In these singly fertilized ovules, to ensure that reproduction can occur, the second synergid cell may attract a second pollen tube, which delivers sperm cells (in our experiments those of GY923 or CAU5) to complete the double fertilization and form type-2 HF or type-3 HF kernels, respectively. Haploid and type-2 HF kernels have the same pigmentation phenotype, meaning that a different phenotype must be used to differentiate between them. We therefore used their oil content (oc) to differentiate between them. In other cases, an ovule with only a fertilized egg cell may have its central cell fertilized during the second fertilization, forming a type-1 HF or type-4 HF kernel. CC, central cell; EC, egg cell; SY, synergid; SYN, synergid nucleus; VCN, vegetable cell nucleus; SC, sperm cell; FCC, fertilized central cell; FEC, fertilized egg cell; DSY, degenerated synergid; PT1, pollen tube 1; PT2, pollen tube 2.

As we shown in Fig. 6, in most cases during haploid induction, double fertilization is the outcome and leads to the formation of colored diploids (~50–70%), while haploids form when chromosome elimination has occurred in the zygote. Singly fertilized ovules may not develop or may develop abnormally to form defective kernels. However, in some cases, an ovule with only a fertilized central cell may develop into a haploid or another pollen tube (in our study from a GY923 or inducer CAU5 pollen) may be attracted by the second (persistent) synergid cell. This second pollination compensates for the first fertilization and forms a type-2 or type-3 HF kernel. We could not classify the type-2 HF kernels according to their pigmentation but could by their OC. Similarly, for ovules with only an egg cell fertilized, a second fertilization by CAU5 or GY923 completes the double fertilization, to form type-4 and type-1 HF kernels, respectively. Type-3 and type-4 HF kernels cannot be uniquely identified from other colored diploids by their pigmentation or by any of their other genetic markers, because their embryos and endosperms come from the same parents, although the sperm cells reach the egg and central cells by different routes; that is, the central cell is the first to be fertilized in type-3 HF kernels while it is the egg cell that is fertilized first in type-4 HF kernels. If the sperm cells released from the first pollen tube remain in a receptive synergid cell rather than fertilizing the egg cell or central cell (Supplementary Fig. S2), ovules may not develop, which results in a decreased rate of seed set. Viable colorless diploids and colored diploids may form if fertilization compensation occurs as described above. Similarly, single fertilization and chromosome elimination after a second fertilization caused by the inducer pollen may lead to the formation of defective kernels and/or haploids.

At present, the observation of maize double fertilization is difficult, so we used an indirect system and present percentages of hetero-fertilization and single fertilized ovules, which provide support for the involvement of single fertilization in haploid induction. However, direct evidence is still required, and the development of breeding lines with fluorescent sperm cells and with haploid-inducing ability, coupled with observations of dynamic double fertilization will help to elucidate the haploid induction mechanism.

### Methods to generate HF kernels

In commercial maize breeding systems, marker-assisted selection, which employs DNA-based genotyping of endosperm, is often used in conjunction with DH technology. However, a mismatch between endosperm and embryo genotypes can affect the accuracy of the genotyping. By checking seven *F*_2_ populations and four three-way crossed populations, Gao *et al.* (2011) found that the average HF rate for these populations was 1.46%, which indicated that the effects of mismatches caused by HF on the accuracy of marker-assisted selection were negligible. Although HF is an unwelcome event in breeding programs, it is useful for research on double fertilization and the interplay between embryo and endosperm (Nagasawa *et al.*, 2013). HF has been reported to occur in angiosperms (Sprague, 1929; Maruyama *et al.*, 2013; Nagasawa *et al.*, 2013), but its frequency is so small that it is of little use in research. Hence, methods to increase the frequency of HF are needed.

In our current study, we detected substantial increases in type-1 and type-2 HF rates in the C5+GY923 pollination according to the *R1-nj* pigmentation and OC reporting systems, whereas we could not identify putative type-3 and type-4 HF kernels because they are physically and genetically identical to colored diploid kernels. Ignoring the possible number of type-3 and type-4 HF kernels, which could affect our percentage calculations, the maximum percentage of type-1 and type-2 HF kernels (5.70%) was found when the female donor 8701 line was doubly fertilized by CAU5+GY923 pollen in the 2016 BJ experiment. When the maternal donor was doubly pollinated by CAU5+GY923 pollen, the genotype of the maternal donor affected the PHFR, which ranged from 1.44 to 6.14% (Table 3). To the best of our knowledge, the highest HF frequency achieved to date has been 25% (Sprague, 1932). Therefore, when designing experiments to examine HF in maize, choosing a female plant that will generate a large proportion of HF kernels should be considered. The pigmentation of maize kernels has commonly been used to distinguish among the different types, as it has been proved by molecular verification to be accurate in well-expressed germplasms (Liu *et al.*, 2017*a*). We used the OC classification system for the research reported here, which has also been shown to be accurate and efficient in sorting type-2 HF and haploid kernels (Supplementary Table S3). Inclusion of the pigmentation system allowed us to also identify type-1 HF kernels. By using both systems it should be possible to study the interactions between the embryo and endosperm during kernel development.

### Factors affecting HF frequency

The length of a maize silk can be more than 30 cm, and the ovules can be fertilized within 8–24 h after pollination. Kapu and Cosgrove (2010) demonstrated that maize silk elongation was considerably reduced only beyond 12 h after pollination, showing that the silk was not wilted and could respond to another pollination within at least 12 h after the first pollination. Although we detected no significant differences in pollen viability and *in vitro* pollen tube growth between normal and inducer lines, delayed *in vivo* pollen germination of inducers has been demonstrated (Xu *et al.*, 2013). To verify our proposed scheme, a relatively large number of ovules fertilized first by an inducer pollen were required; preliminary experiments were carried out that assessed the effects of a second pollination performed at 0, 1, 2, 3, and 4 h after the first, and slightly more induced kernels were detected at 4 h (data not shown). The dual-pollination method was also applied in our previous work to assess the pollen competitive ability of normal and inducer lines (Xu *et al.*, 2013), and the results showed that most ovules were fertilized by the inducer pollen on ears that had first been pollinated by inducer UH400 and then 3.5 h later by the non-inducer line 1680. However, an excess amount of pollen during the first pollination and a delay of the second pollination will clearly interfere with the formation of type-1 and type-2 HF kernels, as pollen from the first pollination may also be available to act in a second pollination. Kato (1999) performed dual pollinations with a separation of 24 h and found that the percentage of HF kernels in seven maize lines with genetically variant backgrounds was only 0.4%. It was speculated that this frequency was a consequence of the distribution of the pollen of the first pollination on the silks. Given our results and based on experience, it seems that the HF frequency in a dual pollination is mainly affected by the silk length, the amount of pollen applied, and the time interval between the first and second pollinations, which is affected by the competitive differences between the pollens of the male parents. Other additional factors may affect the frequency of HF kernel production, such as the growing environment (Table 3).

### The mechanisms underlying *in vivo* haploid induction in maize

In this study, we demonstrated the involvement of single fertilization in the haploid induction process (Fig. 5), suggesting that single fertilization, in addition to chromosome elimination, contributes to haploid formation (Fig. 6). However, how the singly fertilized ovules develop into haploid kernels and the underlying molecular mechanism(s) remain unclear. Single fertilization events are usually induced by bicellular pollen that harbor a single sperm cell as a consequence of a generative cell division failure (Kato, 1997; Iwakawa *et al.*, 2006; Kim *et al.*, 2008). In Arabidopsis, *CYCLIN DEPENDENT KINASE A1* (*CDKA;1*) controls proliferation of generative cells during male gametogenesis. Loss-of-function mutations in *CDKA;1* were first reported to impede cell-cycle progression in pollen that led to generation of a single sperm cell (Iwakawa *et al.*, 2006). However, later reports showed that a significant proportion of single *cdka;1* pollen delivers two sperm cells to the female gamete, and *cdka;1* was therefore considered to delay, but not prevent, division of the generative cell. When a single sperm cell was delivered from a *cdka;1* pollen tube, the female gamete would be fertilized such that the resulting seeds contained a single embryo or a single endosperm. When two sperm cells were released from a *cdka;1* pollen tube, they fused with the two female gametes, but then karyogamy in the central cell usually failed (Aw *et al.*, 2010). It was speculated that this failure resulted from an immediate mitosis of the fertilized central cell that was not synchronized with that of the sperm cell. *F-box-like 17* (*fbl17*) and *cdka;1* mutants display similar phenotypes. FBL17 was reported to be transiently expressed in the generative cell, where it forms an SCF^FBL17^ complex that targets the CDKA;1 inhibitors KRP6 and KRP7 for proteasome-dependent degradation (Kim *et al.*, 2008). Collectively, defects in the second pollen division may result in a single fertilization and aborted seeds owing to failed karyogamy. Interestingly, the promoter activity of the haploid-induction gene *MTL/ZmPLA1/NLD* was reported to coincide with mitosis II during pollen development (Gilles *et al.*, 2017). In addition, aneuploidy and mixoploidy were observed in a wild type × inducer cross (Gao *et al.*, 2011; Zhao *et al.*, 2013), implying that aneuploid sperm cells may result from abnormal divisions of germ cells during development of inducer pollen.

The haploid-induction gene encodes a pollen-specific patatin-like phospholipase A, which functions in membrane remodeling and in the generation of lipid-derived signaling molecules. When this phospholipase A was truncated at its C-terminal region, protein failed to accumulate in the sperm cells (Gilles *et al.*, 2017; Kelliher *et al.*, 2017; Liu *et al.*, 2017*b*). However, the development of the inducer pollen and its involvement in germination appeared normal, which suggests that this protein is involved in the fusion of the sperm and egg cell, and/or in the suppression of embryo formation by an unfertilized egg (Gilles *et al.*, 2017). We were fortunate to observe ovules with an unfertilized egg cell and a fertilized central cell (endosperm) in the haploid-induction crosses (Fig. 5), which provided direct evidence for the single-fertilization hypothesis and identification of the origins of defective kernels produced during haploid induction.

Although only ovules with the central cell alone fertilized were observed using confocal laser-scanning microscopy, the presence of ovules with the egg cell alone fertilized could be speculated because an increase in the resulting type-1 HF was detected. Ovules with an obvious embryo structure but without endosperm were not observed; this was probably due to the fact that the zygote in maize cannot develop further without the presence of its companion endosperm. Our knowledge of gamete adhesion, fusion, and activation in maize is very limited (Zhou *et al.*, 2017). RNA sequencing has shown that certain genes involved in Ca^2+^ signaling and in endomembrane transport and signaling are differentially expressed in inducers and their near-isogenic lines that do not have the haploid-induction gene (Kelliher *et al.*, 2017). We therefore assume that *MTL/ZmPLA1/NLD* affects the induced phenotypes by regulating the related genes. HAP2/GCS1 is an ancient and conserved gamete protein associated with the plasma membrane, and a recent study showed that a conformational change in HAP2/GCS1 is critical for gamete fusion (Fédry *et al.*, 2017). It would be interesting to determine whether a direct or indirect interaction between MTL/ZmPLA1/NLD and HAP2/GCS1 occurs and if such an interaction affects cell fusion.

Similar to haploid-inducing phenotypes, the male gametophytic mutant *kokopelli* (*kpl*) frequently undergoes single fertilizations that result in a reduced seed set (Ron *et al.*, 2010). *KPL* and the inversely transcribed gene *ARI14* generate a sperm-specific *cis*-natural antisense RNA that helps regulate double fertilization. FIS-PRC2 is a chromatin-modifying complex involved in gene silencing via H3K27me3 (Köhler, *et al.*, 2012) and it has been reported to take part in polytube blocking system via the central-cell pathway (Maruyama, *et al.*, 2013). As mentioned above, multiple regulation pathways are involved in double fertilization. Other loci, including *qhir8* (Liu *et al.*, 2015), also contribute to haploid formation when *MTL/ZmPLA1/NLD* is mutated. We speculate that the protein product of *qhir8* functions upstream of MTL/ZmPLA1/NLD, but how it functions and interacts with MTL/ZmPLA1/NLD are unknown. Further studies to explore the function of MTL/ZmPLA1/NLD will help to elucidate the molecular and biological mechanisms of haploid induction, and thus increase our knowledge of double fertilization.

## Supplementary data

Supplementary data are available at *JXB* online.

Table S1. Test results for each type of pollination regime.

Table S2. Detection of type-1 and putative type-2 kernel formation.

Table S3. Verification of type-2 HF kernels from diverse genetic backgrounds according to their oil content.

Fig. S1. Image of an unfertilized egg cell at 2 d after pollination.

Fig. S2. Supplementary schematic model for haploid formation in dual-pollination.

## Supplementary Material

supplementary Tables S1-S3 and Figures S1-S2

## Abbreviations:

BJ: Shangzhuang Experimental Station, Beijing
DAP: days after pollination
DH: doubled-haploid
EmAR: embryo-abortion rate
EnAR: endosperm-abortion rate
HF: hetero-fertilization
HN: Sanya Experimental Station, Hainan Island
OC: oil content
PHFR: putative hetero-fertilization rate
PHPR: putative haploid-production rate.
