# Supplementary material for: Hetero-fertilization together with failed egg–sperm cell fusion supports single fertilization involved in in vivo haploid induction in maize
Source: J Exp Bot. 2018 May 11;69(20):4689–701. doi: 10.1093/jxb/ery177 (PMC6137981; doi:10.1093/jxb/ery177)
Supplement: supplementary Tables S1-S3 and Figures S1-S2 [file ery177_suppl_supplementary_fig-and-table.pdf]

Hetero-fertilization along with failed egg-sperm cell fusion supports single fertilization involved in in vivo haploid induction in maize

Xiaolong Tian, Yuanxin Qin, Baojian Chen, Chenxu Liu, Lele Wang, Xingli Li, Xin Dong, Liwei Liu, and Shaojiang Chen

Supplementary File

Table S1. Test results of each pollination type

pollination type	ears	ICD	non-induced	END	EMB	PH	type-1 HF
CAU5-	13	1205	11	782	72	229	1
CAU5+CAU5	21	2690	23	1459	156	465	5
CAU5+GY923	16	1159	3004	703	61	293	56
GY923+CAU5	14	734	3625	419	35	156	10
GY923-	20	-	-	-	-	-	-

Notes: ICD=induced crossed diploids, END=endosperm abortion kernels, EMB=embryo abortion kernels, PH=putative haploids (haploids+type-2 HF), HF=hetero-fertilization kernels.

Table S2. Detection of type-1 and putative type-2 formation

pollination type	Induced kernels	type-1 HF	putative type-2 HF
CAU5+GY923	2538	70	75
GY923+CAU5	1700	16	25

Table S3. Verification of type-2 HF from diverse genetic backgrounds by oil content

putative haploid	oc	2016 HN				2017 BJ			
		P	G	H	HF	P	G	H	HF
8701	≤6	53	44	40	4	47	29	25	4
	＞6	8	5	0	5	12	6	0	6
C7-2	≤5.5	97	60	56	4	143	104	103	1
	＞5.5	14	9	0	9	11	9	0	9
Z58	≤5	141	46	46	0	40	21	20	1
	＞5	22	18	0	18	9	8	0	8
J24	≤5	42	14	12	2	-	-	-	-
	＞5	3	3	0	3	-	-	-	-
Q319	≤5	80	68	66	2	103	71	70	1
	＞5	29	20	0	20	15	15	0	15

Notes: P=planted, G=germinated, H=haploid, HF=hetero-fertilization.

Figure S1. Unfertilized egg cell at 2 DAP.

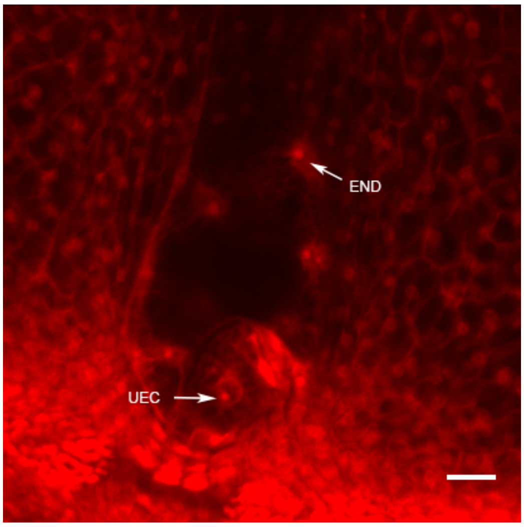

Figure S2. Supplemental model for haploid formation in dual pollination experiment

When neither the egg cell nor the central cell was fertilized in the first fertilization, the second fertilization recovery by sperm cells from GY923, result in colorless diploids (Bo/GY923). If the second pollen tube delivered sperm cells of CAU5, the ovules might be (i) single fertilized, leading to the undeveloped or defective seeds with great probability, while for some ovules with central cell fertilized alone, they had the potential to develop into haploid by parthenogenesis; (ii) or, double fertilized, forming colored diploids with normal development or haploids by chromosome elimination in the subsequent divisions. CC, central cell; EC, egg cell; SY, synergid; SYN, synergid nucleus; VCN, vegetable cell nucleus; SC, sperm cell; FCC, fertilized central cell; FEC, fertilized egg cell; DSY, degenerated synergid; PT1, pollen tube 1; PT2, pollen tube 2.

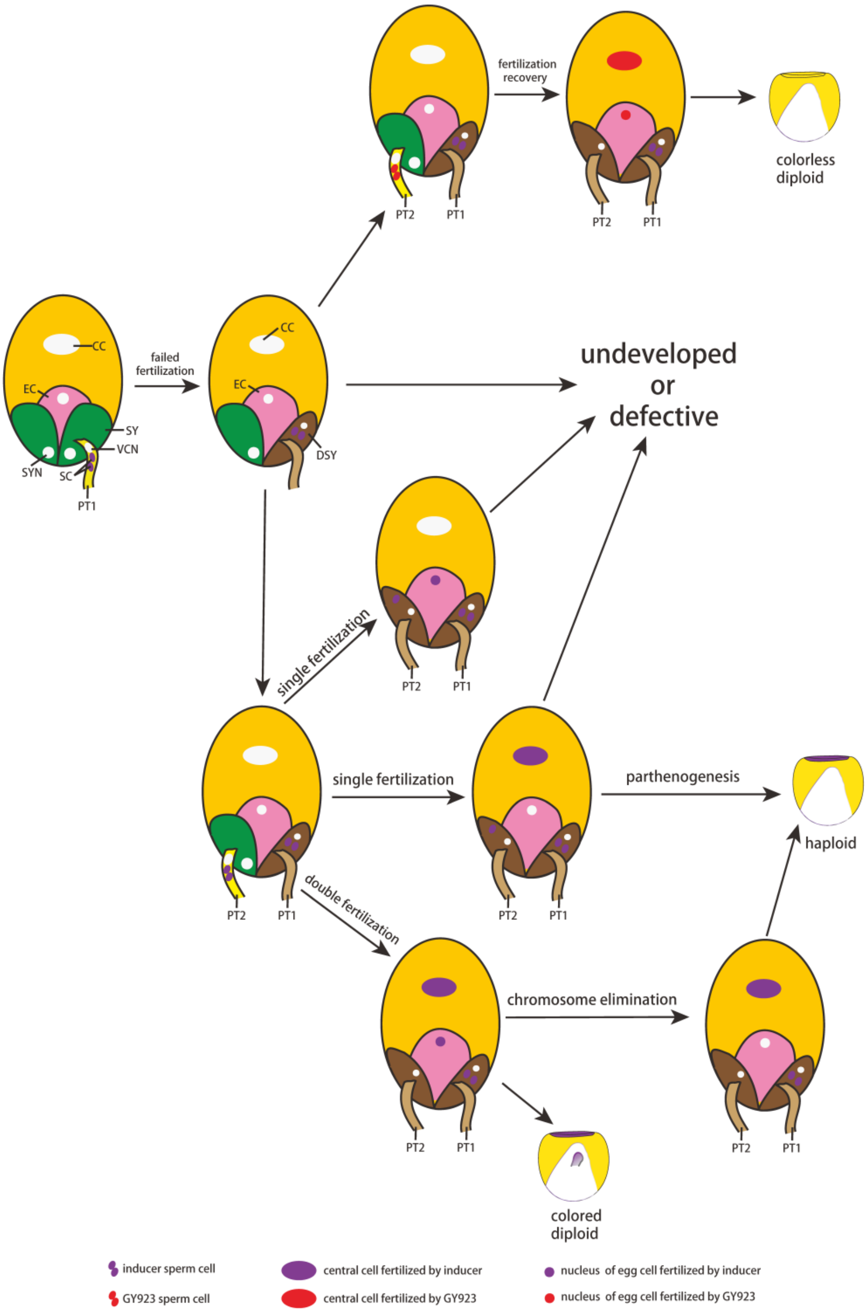

fertilization recovery
colorless diploid
PT2
PT1
CC
EC
SY
VCN
SYN
SC
failed fertilization
DSY
undeveloped or defective
single fertilization
parthenogenesis
haploid
double fertilization
chromosome elimination
colored diploid
inducer sperm cell
GY923 sperm cell
central cell fertilized by inducer
central cell fertilized by GY923
nucleus of egg cell fertilized by inducer
nucleus of egg cell fertilized by GY923
